# Supplementary material for: Boosting Photovoltaic Performance in Organic Solar Cells by Manipulating the Size of MoS2 Quantum Dots as a Hole-Transport Material
Source: Nanomaterials (Basel). 2021 Jun 1;11(6):1464. doi: 10.3390/nano11061464 (PMC8228263; doi:10.3390/nano11061464)
Supplement: Supplementary file 1 [file nanomaterials-11-01464-s001.zip › nanomaterials-1205918-supplementary.pdf]

Supplementary Materials

Boosting Photovoltaic Performance in Organic Solar Cells by Manipulating the Size of MoS_2 Quantum Dots as a Hole-Transport Material

Kwang Hyun Park [1], Sunggyeong Jung [1], Jungmo Kim [2], Byoung-Min Ko [1], Wang-Geun Shim [3], Soon-Jik Hong [1] and Sung Ho Song [1,*]

1 Division of Advanced Materials Engineering, Kongju National University, Cheonan-si 32588, Korea; recite14@gmail.com (K.H.P.); jsk71317@gmail.com (S.J.); qudals3920@gmail.com (B.-M.K.); hongsj@kongju.ac.kr (S.-J.H.)
2 Nano Hybrid Technology Research Center, Korea Electrotechnology Research Institute, 12 Jeongiui-gil, Seongsan-gu, Changwon-si 51543, Korea; jungmokim@keri.re.kr
3 Department of Chemical Engineering, Sunchon National University, 255 Jungang-Ro, Suncheon-si 57922, Korea; wgshim@scnu.ac.kr
* Correspondence: shsong805@kongju.ac.kr; Tel.: +82-041-521-9379

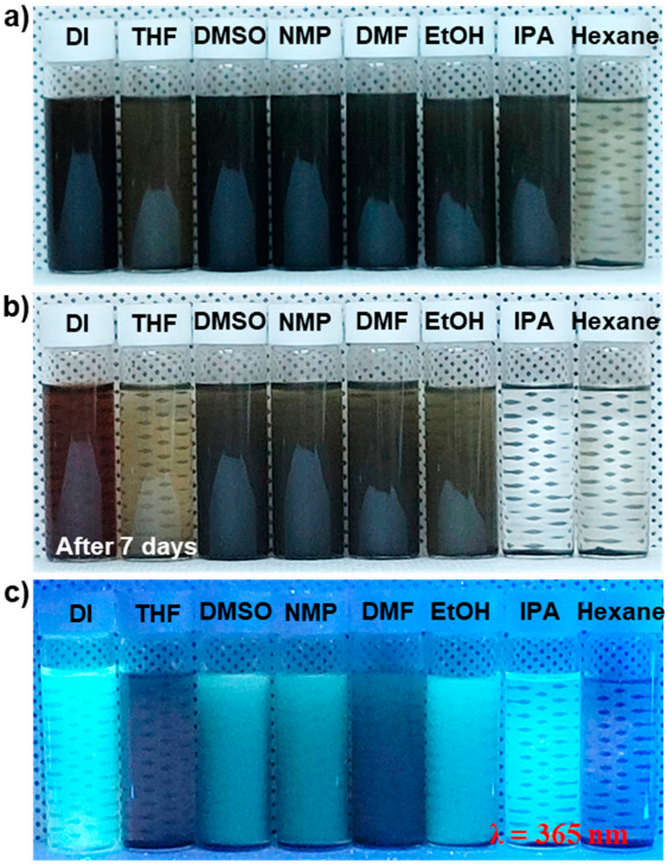

Figure S1. Digital images of the MoS_2 quantum dots (**a**), MoS_2 QDs exfoliated in various solvents with different polarity index. (**b**), MoS_2 QDs dispersed in various solvents after 7 days. (**c**), Photoluminescence of MoS_2 QDs dispersed in various solvents TEM at excitation wavelength of 365 nm.

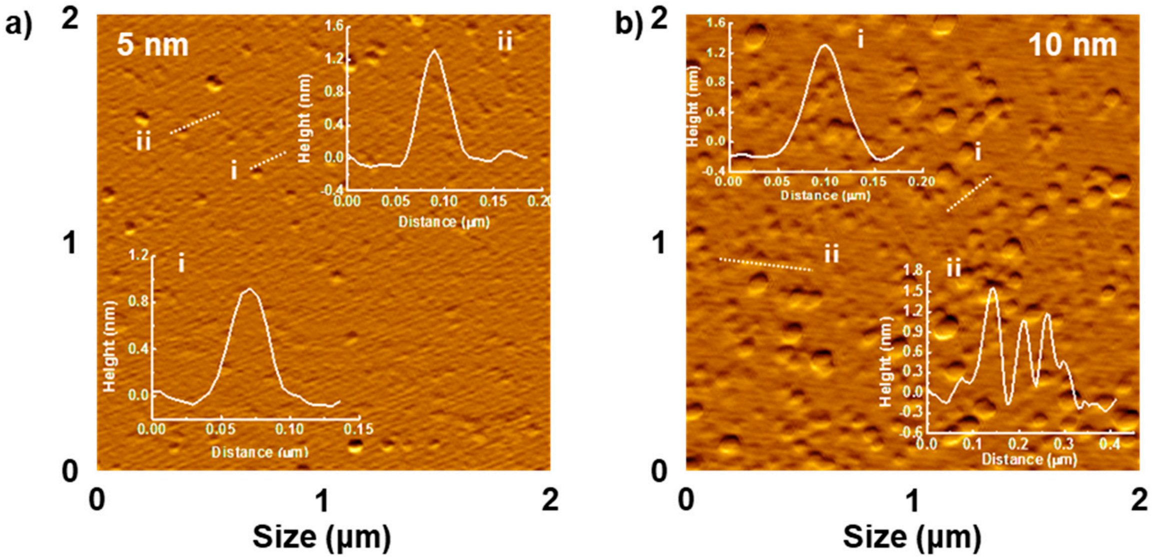

Figure S2. AFM images of 5 and 10 MoS_2 quantum dots (**a**), Topology image of 5 nm MoS_2 QDs. (Inset: thickness profiles of 5 nm MoS_2 QD. (**b**), Topology image of 10 nm MoS_2 QDs. (Inset: thickness profiles of 10 nm MoS_2 QD.

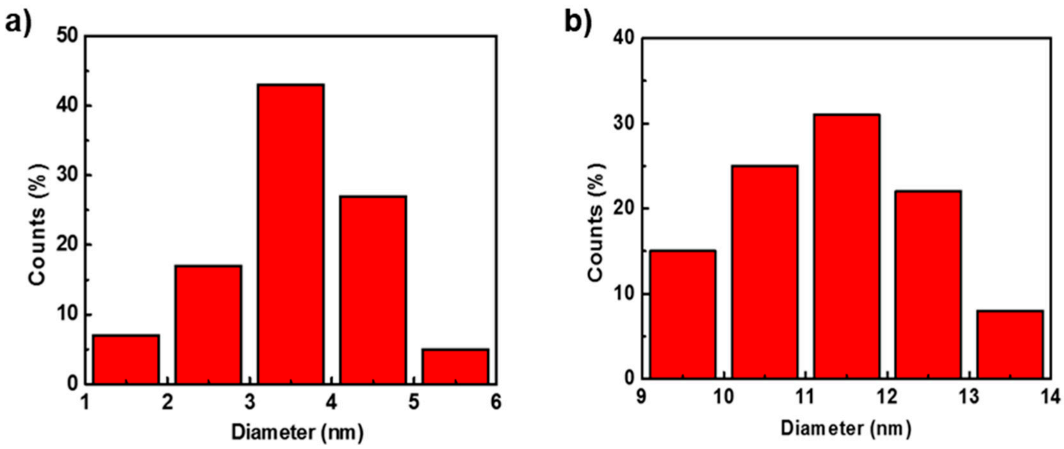

Figure S3. Size distribution of the MoS_2 QDs (**a**) 5 nm MoS_2 QD, (**b**) 10 nm MoS_2 QD.

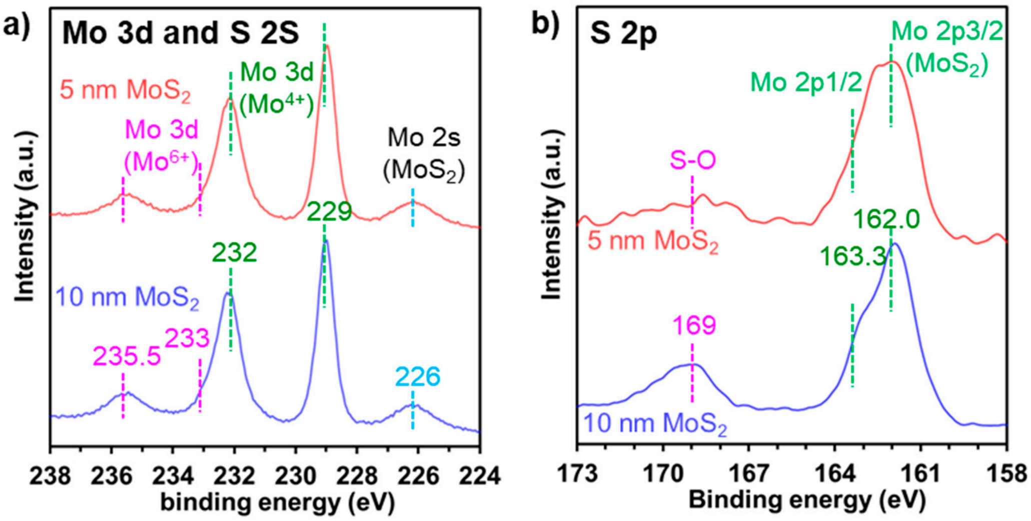

Figure S4. XPS spectra of Mo 3d and S 2p for 5 and 10 MoS_2 quantum dots (**a**), Mo 3d (**b**), S 2p.

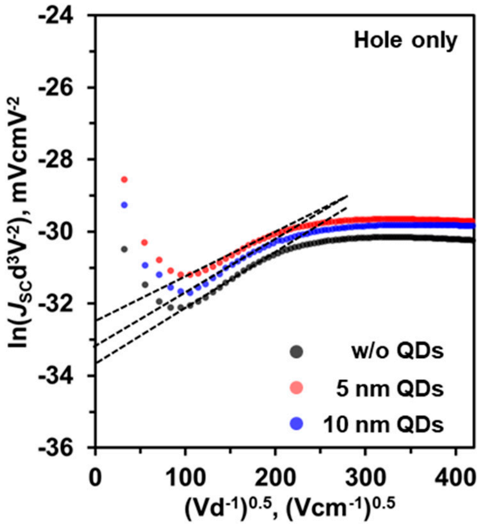

Figure S5. Hole mobility of hole-only device of PTB7:$PC_{71}BM$ with/without MoS_2 QDs by a space charge limited current (SCLC) method.

The hole mobilities for PTB7-based devices fabricated by deposition of 5 nm/10 nm QDs were assessed and estimated by the space-charge limited current (SCLC) method [1,2]. as shown in Figure S4 and Experimental section. The devices fabricated by deposition of 5/10 nm MoS_2 QDs show enhanced hole mobilities ($\mu_h = 2.65 \times 10^{-4}$ cm^2 V^{-1} s^{-1} and $\mu_h = 1.32 \times 10^{-4}$ cm^2 V^{-1} s^{-1} for 5 nm and 10 nm MoS_2 QD, respectively) compared to that of the control device ($\mu_h = 4.2 \times 10^{-5}$ cm^2 V^{-1} s^{-1} for the control device). Especially, the 5 nm MoS_2 QD leads to the highest level of hole mobility, which is consistent with the enhanced performance ascribed to values of J_{SC} and FF in the OSCs.

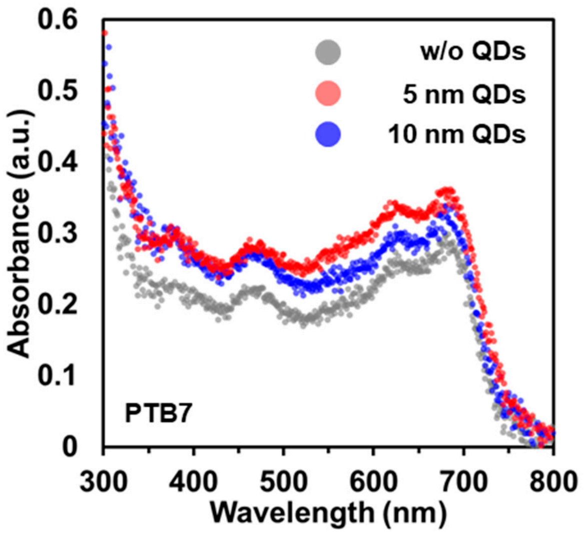

Figure S6. UV-vis absorption spectra of ITO/PEDOT:PSS/hole-transport material/active material films without and with 5 and 10 MoS_2 quantum dots as hole transport layer.

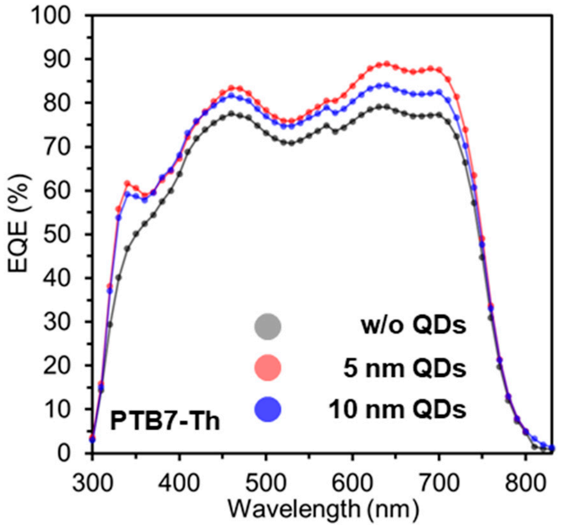

	w/o MoS_2 QD	5 MoS_2 QD	10 MoS_2 QD
J_{SC} (mA/cm^2)	17.9	19.7	19.1

Figure S7. EQE spectra of of PTB7-Th:$PCB_{71}M$ based OSCs with 5 and 10 MoS_2 quantum dot as hole-transport layer.

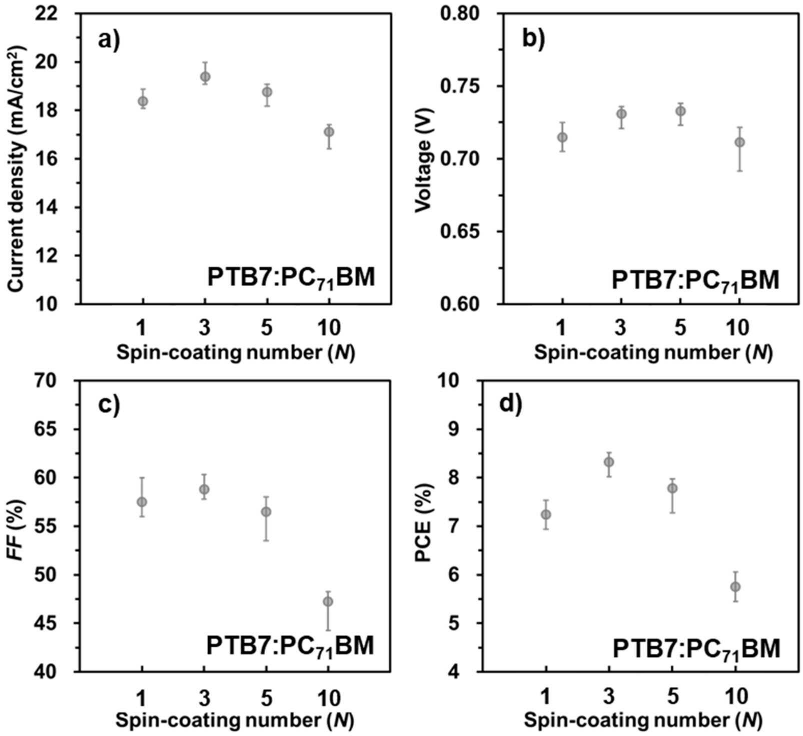

Figure S8. Device characteristics of PTB7:PCB$_{71}$M based OSCs with 5 MoS_2 quantum dot as hole-transport layer. (**a**), short-circuit current density (J_{SC}). (**b**), open-circuit voltage (V_{OC}). (**c**), Fill factor (*FF*). (**d**), Power conversion efficiency (PCE).

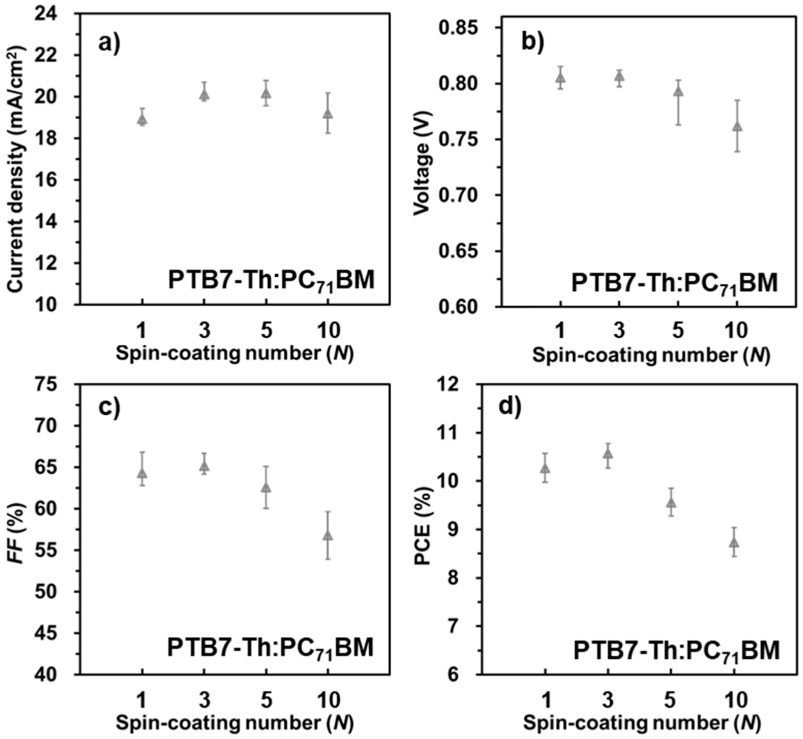

Figure S9. Device characteristics of PTB7-Th:PCB$_{71}$M based OSCs with 5 MoS_2 quantum dot as hole-transport layer. (**a**), short-circuit current density (J_{SC}). (**b**), open-circuit voltage (V_{OC}). (**c**), Fill factor (*FF*). (**d**), Power conversion efficiency (PCE).

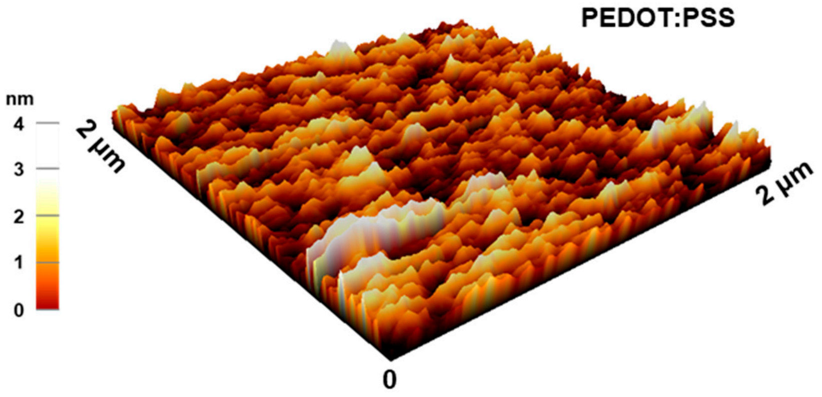

Figure S10. AFM image of ITO/PEDOT:PSS film.

References

1. Sargent, E.H. Colloidal quantum dot solar cells. *Nat. Photon.* **2012**, *6*, 133–135.
2. Sun C.; Qin S.; Wang R.; Chen S.; Pan F.; Qiu B.; Shang Z.; Meng L.; Zhang C.; Xiao M.; et al. High efficiency polymer solar cells with efficient hole transfer at zero highest occupied molecular orbital offset between methylated polymer donor and brominated acceptor. *Am. Chem. Soc.* **2020**, *142*, 1465–1474.
